# Spatial Decomposition of Eddy Covariance Fluxes: The FLUGS Framework

**DOI:** 10.1111/gcb.71031

**Published:** 2026-08-27

**Authors:** Mark Schlutow, Ray Chew, Mathias Göckede

**Affiliations:** ^1^ Department Biogeochemical Signals Max Planck Institute for Biogeochemistry Jena Thuringia Germany; ^2^ Division of Geological and Planetary Sciences California Institute of Technology Pasadena California USA

**Keywords:** Eddy covariance, environmental response functions, flux spatialization, footprint modeling, representativeness, spatial flux decomposition, spatial heterogeneity, upscaling

## Abstract

Understanding how heterogeneous landscapes contribute to ecosystem‐exchange fluxes remains a significant challenge for utilizing eddy covariance (EC) measurements. This work presents FLUGS, a novel framework that infers land‐cover‐specific ecosystem‐exchange fluxes provided the EC time series of aggregated fluxes and the land cover map of the ecosystem. Using a multitask machine learning approach based on Kernel Ridge Regression combined with high‐resolution flux footprints, FLUGS learns the environmental response functions (ERFs) from EC data for each land cover class simultaneously. FLUGS integrates a state‐of‐the‐art atmospheric transport model that provides accurate, computationally efficient footprint estimates with a statistically rigorous, convex optimization method for submeso‐scale flux inversion. The approach is versatile, robust to multicollinearity and yields smooth and interpretable ERFs with a unique global optimum. We evaluate FLUGS using virtual and physical experiments derived from synthetic data and observational data from an EC site in Northeast Siberia. Across all tests, the framework accurately reconstructs land‐cover‐specific sensible heat and ${\mathrm{CO}}_{2}$ fluxes and captures systematic differences between land cover classes that reflect local hydrological and ecological conditions. FLUGS also provides statistical performance metrics and cross‐tower consistency diagnostics that quantify uncertainty in heterogeneous landscapes. By offering a fast, transparent workflow for spatially decomposing ecosystem fluxes, FLUGS opens new opportunities to attribute EC fluxes to ecological processes, benchmark land‐surface models and improve our understanding of land‐atmosphere interaction.

## Graphical abstract

Understanding how heterogeneous landscapes shape ecosystem‐exchange fluxes remains a major challenge for interpreting eddy covariance (EC) measurements. We present the FLUGS framework that infers land‐cover‐specific fluxes from aggregated EC time series and land‐cover maps. FLUGS combines multitask kernel ridge regression with high‐resolution flux footprints to simultaneously learn land‐cover‐specific environmental response functions. Tests using synthetic data and EC observations show that FLUGS accurately reconstructs sensible heat and CO_2_ fluxes and captures systematic differences among land‐cover classes linked to hydrological and ecological conditions. FLUGS also provides uncertainty metrics and diagnostics, enabling robust flux attribution in heterogeneous landscapes.

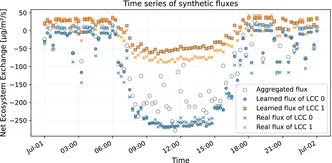

## 1 Introduction

Continuous monitoring of energy and greenhouse gas turbulent exchange fluxes between the biosphere and atmosphere using the eddy covariance (EC) method is an instrumental tool to understand Earth's climate system (Baldocchi 2020). The application of EC is based on a number of theoretical assumptions, a key one being the requirement of spatial homogeneity within the field of view (or footprint) of the tower‐mounted instruments (Göckede et al. 2004; Chu et al. 2021). This assumption, however, is often violated, since the majority of EC sites are situated in complex terrain such as mixed forest stands, wetlands, mires, permafrost peatlands, or urban landscapes (Göckede et al. 2008; Crawford and Christen 2015; Levy et al. 2020; Moutahir et al. 2026; Yazbeck et al. 2025).

Heterogeneity within an EC tower footprint complicates the interpretation of measured fluxes, because the observed net flux integrates signals from multiple small‐scale land cover (LC) elements with often distinct flux signatures. As a result, the representativeness of EC fluxes for upscaling is limited to ecosystems with similar LC composition. Training and evaluating land surface models (LSMs) is also affected, since their parameter estimation commonly relies on flux time series from EC towers (Thum et al. 2019; Lawrence et al. 2019). When footprints are heterogeneous, parameters that govern the surface energy budget or terrestrial carbon cycle for a single LC type may be biased, because the training data reflect surface‐atmosphere exchange from a mixed ecosystem. Ensuring the representativeness of EC fluxes is therefore essential for LSM development, and biased training may degrade the quality of simulations of the current climate and reduce the reliability of future climate projections.

A promising approach to address this issue is spatial flux decomposition, that is, attributing aggregated flux measurements to individual spatial features in a heterogeneous landscape. Existing methods rely on the key assumption of ecosystem similarity, meaning that patches within the same land cover class respond similarly to temporal changes in environmental controls. Wang et al. (2006) introduced this idea for EC data by combining footprint simulations with a process‐based ecosystem model consisting of closed‐form environmental response functions (ERFs) for photosynthesis and soil respiration. Similar techniques were applied in an urban setting by Crawford and Christen (2015), who extended the ERFs to combustion processes, and by Moutahir et al. (2025), who used a sophisticated process‐based terrestrial ecosystem model. Additional decomposition approaches for methane that did not require ERFs because of sufficiently steady fluxes were presented by Tuovinen et al. (2019) and Rey‐Sanchez et al. (2022). All of these approaches, however, remain limited by their degrees of freedom, addressing only specific flux types, ecosystems, or land cover uses.

Lately, several modeling frameworks for spatial flux decomposition have been presented that follow a more general strategy and learn the ERFs from the EC data without prior process knowledge, therefore providing more degrees of freedom for capturing deviating responses from various ecosystem elements. Xu et al. (2017) generalized the ERF approach for the EC method to a data‐driven machine learning task. They utilized Boosted Regression Trees (BRTs), which have certain benefits for learning ERFs. BRTs handle multicollinear environmental drivers well, i.e., drivers that are correlated like temperature and net radiation. However, they are prone to discontinuities as they only predict step functions. As ERFs in nature are continuous, this behavior may result in unrealistic predictions. Furthermore, BRT may converge to a local optimum in parameter space, providing plausible ERFs but not necessarily the best (i.e., the global optimum).

Levy et al. (2020) inferred land‐cover‐specific methane and sensible heat fluxes using a Bayesian linear regression as a machine learning tool, but their study is limited to linear ERFs that are unable to represent nonlinear phenomena like saturation and photoinhibition. Pirk et al. (2024) employ a Bayesian Neural Network (NN) to spatially disaggregate carbon exchange of degrading permafrost peatlands. Their NN approach allows for a relatively large number of environmental drivers to be used for ERF learning. However, NNs are notoriously difficult to interpret and also prone to discontinuous responses. Like BRT, NNs do not always converge to the global optimum and find the best ERFs.

In this work, we introduce FLUGS, a novel and versatile framework for spatial flux decomposition that improves upon recent ideas while also addressing the shortcomings of previous approaches:

1. Footprints are computed with the improved Boundary Layer Dispersion and Footprint Model (BLDFM) for atmospheric transport, which employs modern, fast and accurate numerical methods (Schlutow et al. 2026)
2. The assumptions FLUGS makes are minimal; apart from the steady‐state assumption in BLDFM and the assumptions of the turbulent model, no other process knowledge is required
3. A new machine learning method infers land‐cover‐specific ERFs based on multitask Kernel Ridge Regression (KRR), which is broadly applicable across different flux types, ecosystems and land cover uses. In contrast to BRTs and NNs, it always finds the unique global optimum and predicts continuous ERFs
4. FLUGS provides advanced statistical metrics to evaluate its accuracy and performance
5. The model is well tested against virtual synthetic experiments and real‐life observational data

As such, spatial flux decomposition with FLUGS is particularly applicable for heterogeneous landscapes, when the flux signals from the different LC elements exhibit a significant spread. One week of high‐quality flux and meteorological data suffices to accurately train FLUGS with two land cover classes. More classes require more available data. The decomposition's accuracy is mainly determined by footprint contrast, flux data filtering, and the availability and quality of the environmental drivers.

## 2 Methods

**FIGURE 1:**
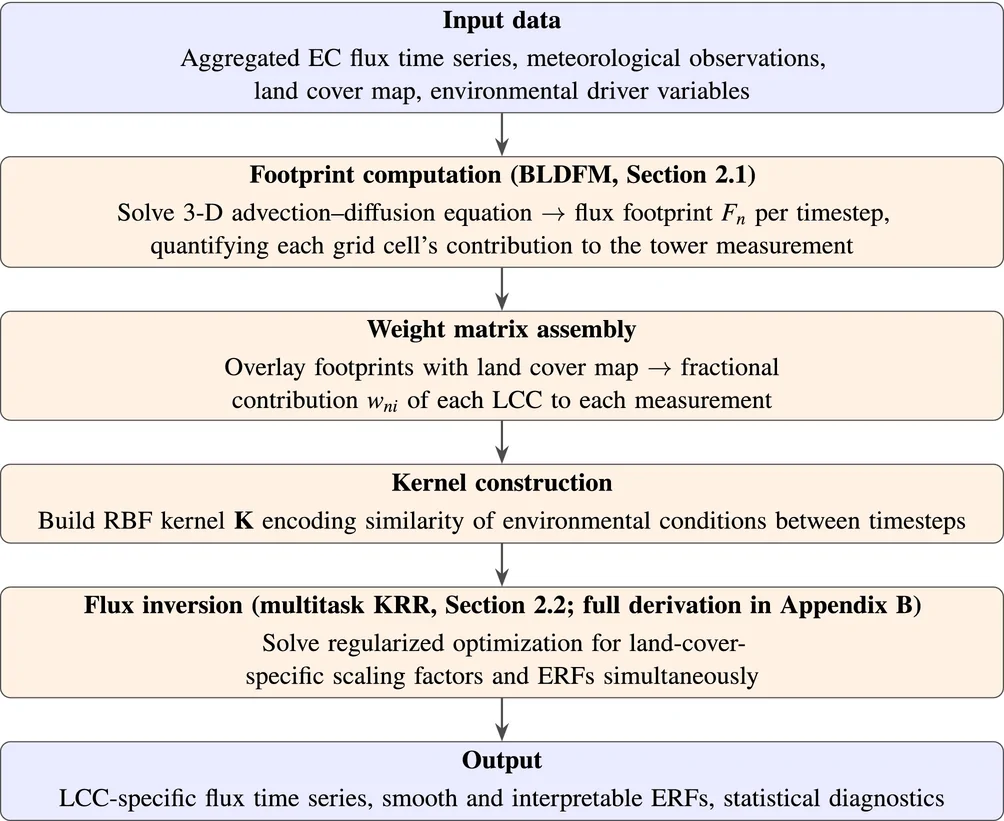
Overview of the FLUGS workflow. Input data are processed through footprint computation, weight matrix assembly, and kernel construction before the multitask kernel ridge regression solves for land‐cover‐specific fluxes and environmental response functions. See Section 2.2 and Appendix B for the full mathematical derivation.

Figure 1 provides an overview of the FLUGS workflow, described in detail below.

### 2.1 The Atmospheric Transport Model

A core component of FLUGS is the Boundary Layer Dispersion and Footprint Model (BLDFM) (Schlutow et al. 2026), a fast numerical solver for the three‐dimensional steady‐state advection–diffusion equation in Eulerian form. As a hybrid model, it can operate in either dispersion mode or footprint mode. It takes sub‐hourly meteorological EC data as input, including wind speed as well as direction, roughness length, friction velocity and the Monin‐Obukhov length, from which vertical profiles of mean‐flow horizontal wind as well as eddy diffusivity are computed via Monin‐Obukhov Similarity Theory. Depending on the mode, BLDFM outputs concentration and flux fields or their corresponding footprints. The model is available as an open‐source software package^1^ (Schlutow and Chew 2025a).

### 2.2 Flux Inversion With Multitask Multivariate Kernel Ridge Regression

A second key component of FLUGS is the flux inversion formulated as an optimization problem. Let i=1,2,…,I index nonoverlapping land cover classes (LCCs), for example, grass and shrubs. Each LCC is associated with a spatial mask

(1) $$m_{i}\left(x,y\right)=\left\{\begin{array}{l} 1\;\text{if}\;\left(x,y\right)\in {\mathrm{LCC}}_{i},\\ 0\;\text{else}, \end{array}\right.$$

such that each surface location $\left(x,y\right)$ belongs uniquely to one LCC. The surface flux at time index n=1,2,…,N is represented as

(2) $$\begin{array}{r} q_{0n}\left(x,y\right)=\sum_{i=1}^{I}m_{i}\left(x,y\right)\;a_{\text{in}}, \end{array}$$

where the unknown coefficients $a_{\text{in}}$ are time‐dependent scaling factors that represent the surface flux associated with each LCC (Rödenbeck et al. 2018). Using BLDFM in footprint mode, the flux measured at $\left(x_{M},y_{M},z_{M}\right)$ at time n is given by

(3) $$\begin{array}{r} q_{n}\left(x_{M},y_{M},z_{M}\right)=\sum_{x,y}F_{n}\left(x_{M},y_{M},z_{M};x,y\right)\;q_{0n}\left(x,y\right). \end{array}$$

Inserting (2) into (3) and exploiting linearity yields

(4) $$\begin{array}{r} q_{n}=\sum_{i=1}^{I}w_{\mathrm{ni}}a_{\text{in}}, \end{array}$$

with flux footprint weights

(5) $$\begin{array}{r} w_{\mathrm{ni}}=\sum_{x,y}F_{n}\left(x_{M},y_{M},z_{M};x,y\right)\;m_{i}\left(x,y\right). \end{array}$$

The predictive model Equation (4) forms the basis of the flux inversion.

Because the scaling factors vary in time, they are modeled as functions of environmental driver variables measured at the EC tower, such as air temperature, humidity and incoming shortwave radiation. These drivers are assumed spatially homogeneous across LCCs at a given time. The resulting functional relationships are referred to as Environmental Response Functions (ERFs; Metzger et al. 2013; Xu et al. 2017). While parametric ERFs are commonly used for specific fluxes as well as ecosystems, they require prior assumptions about functional form and parameters.

Here, FLUGS adopts a nonparametric multitask learning approach based on Kernel Ridge Regression (Bishop 2006), learning ERFs for all LCCs simultaneously without prescribing ecosystem‐specific structure. Through the kernel function, KRR captures nonlinear relationships between driver variables and fluxes, including phenomena such as exponential temperature dependence, without prescribing a functional form. This makes the framework broadly applicable across flux types and ecosystems, with the choice of driver variables determined by the user.

In matrix notation, with $A=\left(a_{\text{in}}\right)$ and $W=\left(w_{\mathrm{ni}}\right)$, the solution for the scaling factors is

(6) $$\begin{array}{r} A=W^{T}\mathrm{diag}\left({\left(K\circ WW^{T}+\lambda I_{N}\right)}^{-1}\;\hat{q}\right)K. \end{array}$$

A detailed derivation is provided in Appendix B. The operator $\mathrm{diag}\left(\cdot \right)$ takes in a N vector and returns a N×N matrix of mostly zeros with the vector elements on the diagonal. The symbol ∘ denotes the Hadamard product, i.e., the element‐wise multiplication of two matrices of the same size, and the symbol T transposes a matrix. The regularization parameter λ mitigates the effects of collinearity among driver variables.

Environmental drivers enter through the kernel matrix K, defined using a radial basis function (RBF) kernel or Gaussian kernel function,

(7) $$\begin{array}{r} {\left(K\right)}_{\mathrm{nm}}=\exp\left(-\frac{∥x_{n}-x_{m}{∥}^{2}}{2l^{2}}\right), \end{array}$$

where $x_{n}$ is the vector of normalized driver variables at time n, and l is a hyperparameter controlling the smoothness of the ERFs. Finally, $\hat{q}$ denotes the vector of measured EC fluxes.

In summary, FLUGS requires only three assumptions to be met to successfully decompose fluxes spatially:

- *Spatial homogeneity of drivers*: Environmental conditions (e.g., temperature, solar radiation) are uniform across all patches
- *Ecosystem stationarity*. The ecosystem's baseline state remains stable, assuming no major changes (such as ecosystem disturbances, land management or ecological succession) alter its function during the training phase
- *Ecosystem similarity*. Every patch within the same LCC has identical biogeochemical flux response to any given environmental change

### 2.3 Validation and Application of FLUGS

#### 2.3.1 Corroboration by Synthetic Data in a Virtual Experiment

Spatial flux attribution with FLUGS is not a canonical supervised machine learning task, as the ground truth, i.e., the surface fluxes per LCC, which would be used for training, is unknown. To corroborate the performance of FLUGS, we therefore designed a synthetic simulation experiment that mimics a real EC tower with the difference that the surface fluxes for each LCC are predetermined.

A suitable land cover map with two distinct LCCs is stochastically generated using Gaussian Random Fields as described in Schlutow et al. (2024) with the Python software package GSTools (Müller et al. 2022). Each LCC is associated with a flux given by a parametric ERF for Net Ecosystem Exchange (NEE) based on Runkle et al. (2013). The ERF depends on the photosynthetically active radiation (PAR) and temperature as environmental driver variables that are prescribed by a typical diurnal cycle. As PAR and temperature are highly correlated, this setup is chosen to be particularly challenging. To test the accuracy of FLUGS, white Gaussian noise at 10% amplitude of the signal is added to the environmental variables. The precise definition of the ERF function, together with the parameters that determine the individual surface fluxes as a reaction to the drivers and the definition of the diurnal driver variables, is provided in Appendix C.

For the simulation of atmospheric transport, BLDFM is utilized in dispersion mode. A neutrally stable boundary layer is assumed with 0.6 m s^−1^ friction velocity and winds blowing from random directions with wind speeds between 0 and 5 m s^−1^ drawn from a uniform random distribution. BLDFM predicts the flux field under these meteorological conditions. The aggregated flux is measured from the resulting simulated flux field by a virtual eddy tower at 5 m height. To make the experiment more realistic and mimic measurement uncertainty, white Gaussian noise at 10% amplitude of the signal is also added to the aggregated flux.

Subsequently, FLUGS is given the stochastic land cover map, the time series spanning 24 h of meteorological data and noisy environmental drivers, and the time series of the noisy point measurement of the simulated ${\mathrm{CO}}_{2}$ flux. From this noisy and sparse data set, FLUGS attempts to decompose the fluxes spatially into their individual components originating from the two different LCCs.

#### 2.3.2 A Case Study: The Cherskii Eddy Covariance Towers

Our study site is the Ambolikha research site (68°36′46″ N, 161°21′27″ E) in Northeast Siberia near the town of Chersky (Kittler et al. 2016; Göckede et al. 2017). The site is underlain by continuous permafrost and is classified as wet tussock tundra, forming a highly heterogeneous ecosystem.

Since 2013, two identically instrumented eddy covariance (EC) towers with measurement heights of approximately 5 m above ground level have been operated as part of a disturbance experiment. One tower (RU‐Che) is located within an artificial drainage ring designed to study the effects of a lowered water table on a formerly wet permafrost ecosystem, while the second tower (RU‐Ch2) is situated in an undisturbed control area. Raw data from both towers were processed using the EddyPro software.^2^ Details on instrumentation, data processing, as well as quality assessment are provided in Bolek et al. (2025).

Because both sites use identical instrumentation and processing workflows, the experimental design excludes systematic methodological differences between the observational data sets. The Cherskii EC towers therefore provide an ideal test case for FLUGS. Both towers sample the same ecosystem components within their footprints, but with different fractional land‐cover compositions. Spatial flux decomposition should thus yield consistent land‐cover‐specific fluxes across towers, despite differences in their net flux measurements. Comparing the ground‐level, land‐cover‐class‐specific fluxes derived by FLUGS is the objective of this experiment.

The land cover classification for the Ambolikha sites is based on two WorldView2 scenes acquired in July and August 2011 at 2 m spatial resolution. Following atmospheric correction using preselected regions of interest, a minimum distance classification was performed and validated against an in situ calibration dataset. The resulting classes were simplified to 14 major land cover types distinguished primarily by vegetation structure and height. For this study, the classification was further aggregated into two categories: short vegetation (grass, G) and tall vegetation (shrubs and trees, S + T).

### 2.4 Statistical Metrics: Scoring FLUGS

The quality of FLUGS' decomposition is evaluated in terms of the coefficient of determination ($R^{2}$), the Durbin and Watson (1950) test for autocorrelation of the residuals as well as the Shapiro and Wilk (1965) test for normality of the residuals.

The Durbin‐Watson (DuWa) test produces a statistic ranging from 0 to 4, where a value around 2 indicates no autocorrelation, values approaching 0 suggest positive autocorrelation, and 4 suggests negative autocorrelation of the residuals. This test verifies the independence assumption of the regression and identifies whether the model may omit relevant environmental drivers.

Ridge regression assumes normality of the residuals. The Shapiro–Wilk (ShWi‐p) test provides a *p*‐value to evaluate whether model residuals are drawn from a normally distributed population. This *p*‐value refers to the null hypothesis that the data are normally distributed, and the hypothesis is rejected if it falls below a predetermined significance level.

The subsequent section will refer to these metrics, with $R^{2}=1.0$, DuWa = 2.0 and ShWi‐p > 0.01 as the optimal scores.

## 3 Results and Discussion

### 3.1 Virtual Experiment With Synthetic Data

**FIGURE 2:**
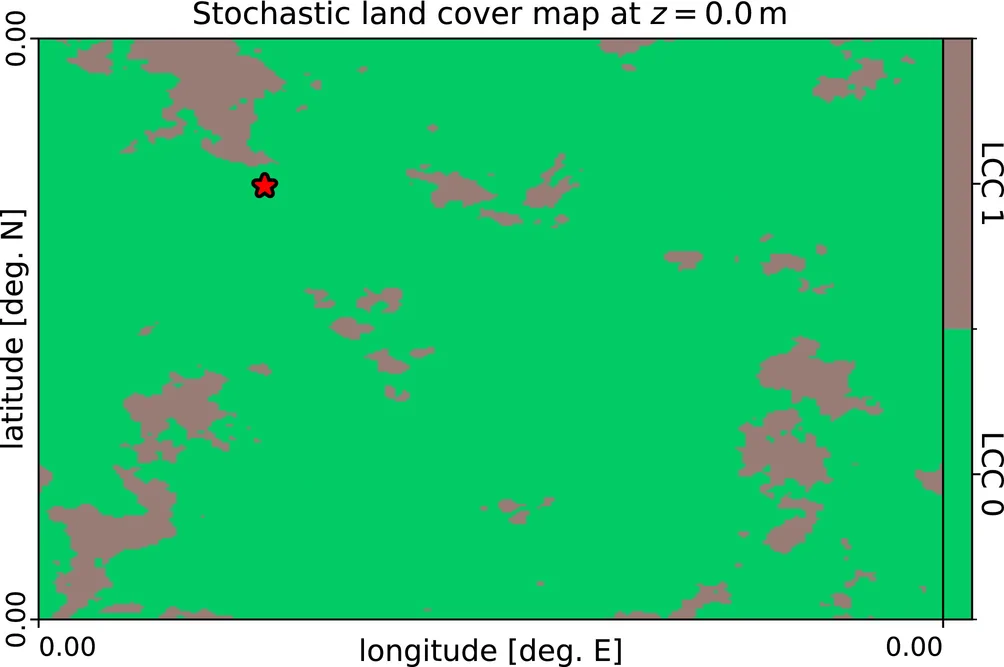
Stochastic land cover map with arbitrary two land cover classes, LCC 0 (green) and LCC 1 (brown). The red star marks the EC measurement tower location.

The randomly generated stochastic land cover map with two LCCs comprising $\left(256\times 256\right)$ grid points is shown in Figure 2. Note that the domain extents are arbitrary and do not affect the subsequent results. The marker corresponds to the location of the virtual EC tower at grid‐point index $\left(64,192\right)$.

**FIGURE 3:**
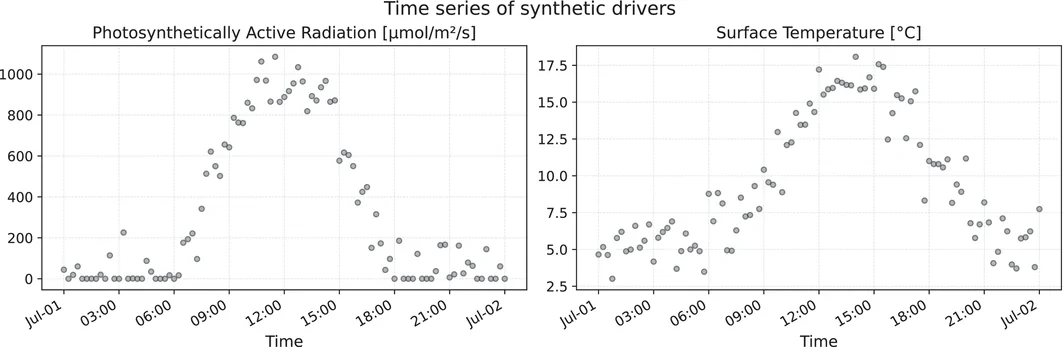
Time series of the synthetic driver variables; see Appendix C and the accompanying text for more details.

Time series of artificially generated noisy driver variables are shown in Figure 3. An arbitrary start time of July 1st 00:00 h is used, and the synthetic data are generated over 1 day with a 15 min interval; see Appendix C for more details. The BLDFM footprints are computed with $\left(256\times 256\right)$ spectral modes and 32 discrete vertical grid cells. For KRR, an RBF kernel is used with a length scale of l=0.6 and a regularization factor of λ=0.1.

**FIGURE 4:**
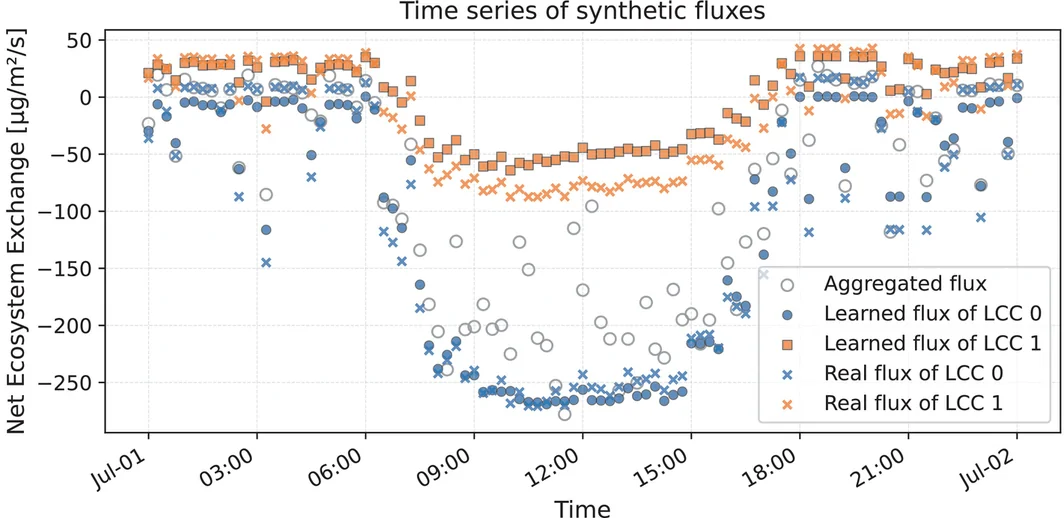
Time series of FLUGS‐computed fluxes for LCC 0 (blue circles) and LCC 1 (orange squares) compared with the known reference fluxes (crosses). The measured aggregated flux is shown for reference (gray circles). Close agreement between learned and reference fluxes demonstrates successful recovery of land‐cover‐specific fluxes from a single aggregated measurement despite 10% measurement noise.

Finally, Figure 4 shows the aggregated as well as the reference LCC fluxes along with the learned outputs from FLUGS. Despite sampling only aggregated measurement data at one spatial location, FLUGS can reconstruct the fluxes per LCC to a large extent. Specifically, the FLUGS‐computed fluxes (solid circles and squares for LCC 0 and 1, respectively) closely reproduce the real, unobserved, LCC fluxes (crosses). Quantitatively, the statistical scores are $R^{2}=0.973$, DuWa=1.895 and $\text{ShWi}-p=2.464\times 10^{-4}$. The $R^{2}$ and DuWa scores suggest a high‐quality model decomposition, indicating that the model explains the vast majority of the variance and the residuals are independent without any significant autocorrelation.

**FIGURE 5:**
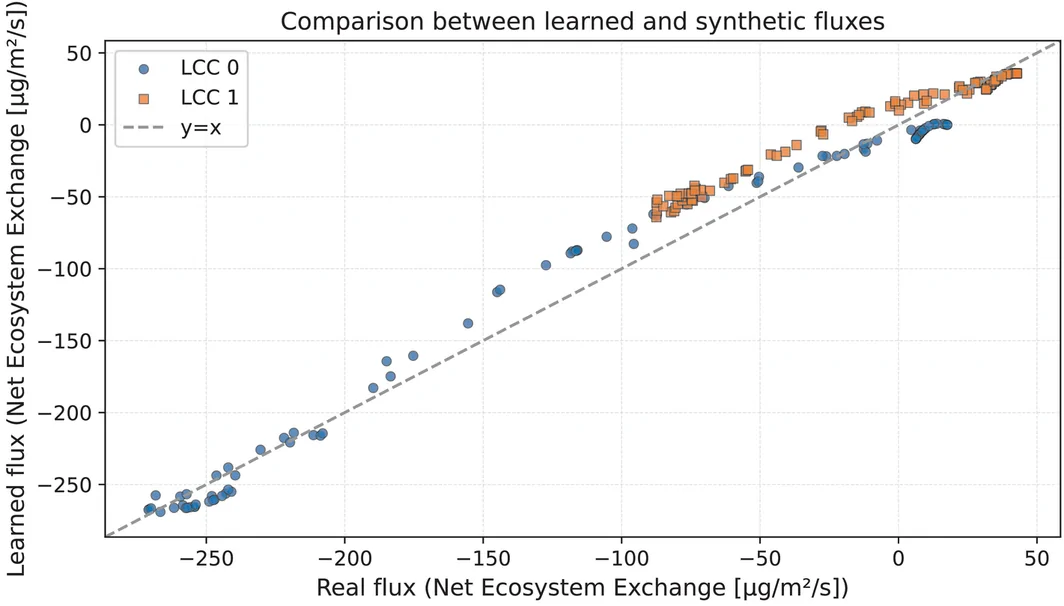
Scatter plot of learned versus reference fluxes for LCC 0 (blue circles) and LCC 1 (orange squares). The dashed line indicates perfect agreement (y=x). Points cluster tightly around this line ($R^{2}=0.973$), confirming quantitative accuracy of learned fluxes to reproduce the true values. Sparse sampling at intermediate flux values produces the slight systematic spread visible near the center of the range.

The low ShWi‐p value is likely a consequence of data sparsity rather than a model deficiency. In particular, the distribution of flux values is dense near the two ends of the NEE range but very sparse at intermediate values, as seen in Figure 5. This uneven sampling creates an empirical residual distribution that deviates from normality, which leads to a statistically significant rejection by the Shapiro–Wilk test. Importantly, this effect is consistent with the bias‐variance dilemma: when the dataset is small or unevenly sampled, KRR yields low variance but a higher degree of bias in regions with few observations. The low ShWi‐p value, therefore, should not be interpreted as evidence of overfitting. Instead, it indicates that FLUGS can reliably recover fluxes even when the underlying dataset is sparse.

To visualize the overall accuracy of the decomposition, we compare the learned fluxes directly against the known reference values in a scatter plot.

**FIGURE 6:**
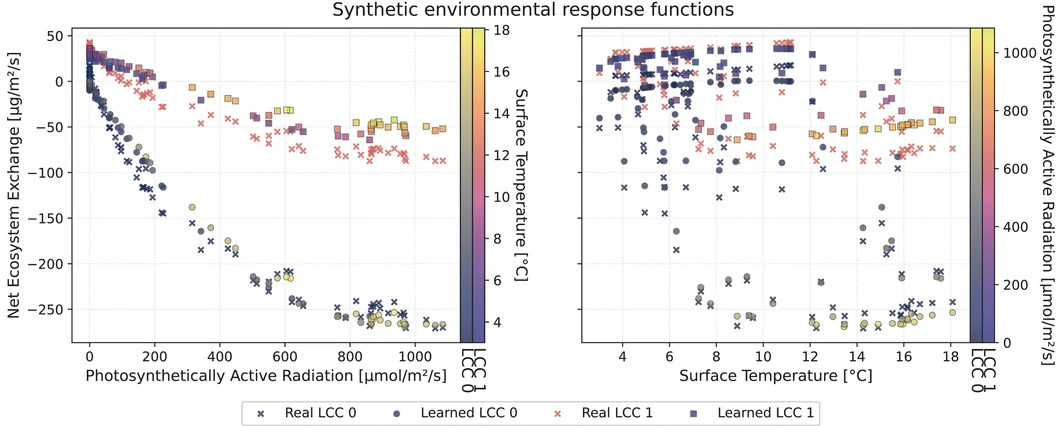
Comparison of reference ERFs (crosses) and learned ERFs (solid markers) for LCC 0 (circles) and LCC 1 (squares). Left: Flux response to PAR, showing the nonlinear saturation correctly captured by FLUGS. Right: Flux response to surface temperature. Marker colors indicate the value of the secondary driver variable (colorbars). The close overlap confirms that FLUGS infers realistic driver–flux relationships without prescribing a parametric form and is largely capable of recovering the true environmental response functions.

The ability of FLUGS to capture the functional relationships between driver variables and fluxes per LCC is illustrated in Figure 6. The real ERFs (crosses) are compared to the learned ERFs (solid circles and squares). For both LCCs, the learned ERFs reproduce the dependencies of the real fluxes on PAR and surface temperature (T) to a large degree. In particular, the left panel shows that the model correctly identifies the strong nonlinear saturation of ${\mathrm{CO}}_{2}$ uptake with increasing PAR. The right panel illustrates the ERF's sensitivity to T as modulated by PAR levels, where the data points largely group into two clusters corresponding to low‐PAR/high‐T and high‐PAR/low‐T conditions.

Minor deviations between the learned and real ERFs, especially at intermediate driver values, reflect the limited number of samples available in this region of the driver space. When data density is low, KRR necessarily adopts a smoother representation of the ERF, which can introduce small systematic offsets relative to the true response. Despite this low data density, FLUGS nevertheless captures the dominant environmental sensitivities governing the aggregated flux with high fidelity, and the agreement across LCCs demonstrates that the method can recover realistic, class‐specific response functions even from temporally sparse and spatially aggregated datasets.

Model sensitivity is further investigated in Appendix D where parameter sweeps over the noise level and number of LCCs are performed and analyzed.

### 3.2 Experiment With Observational Data From the Cherskii Site

**FIGURE 7:**
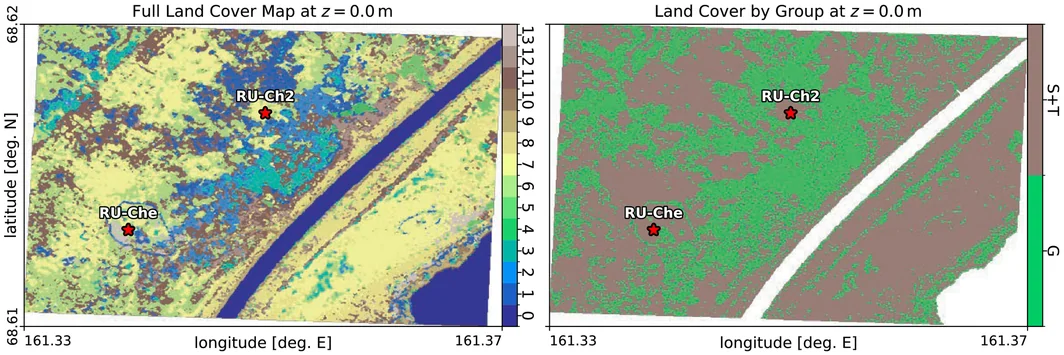
Land cover map of the Cherskii site: Processed WorldView2 data with 14 land cover classes (left) and grouped by “grass” as well as “shrubs and trees” (right). The red stars mark the position of the EC towers.

The locations of the two Cherskii towers (red stars in Figure 7) are depicted for the processed WorldView2 scene (left panel) along with the resulting grouped LCCs used in the subsequent analyses (right panel).

**FIGURE 8:**
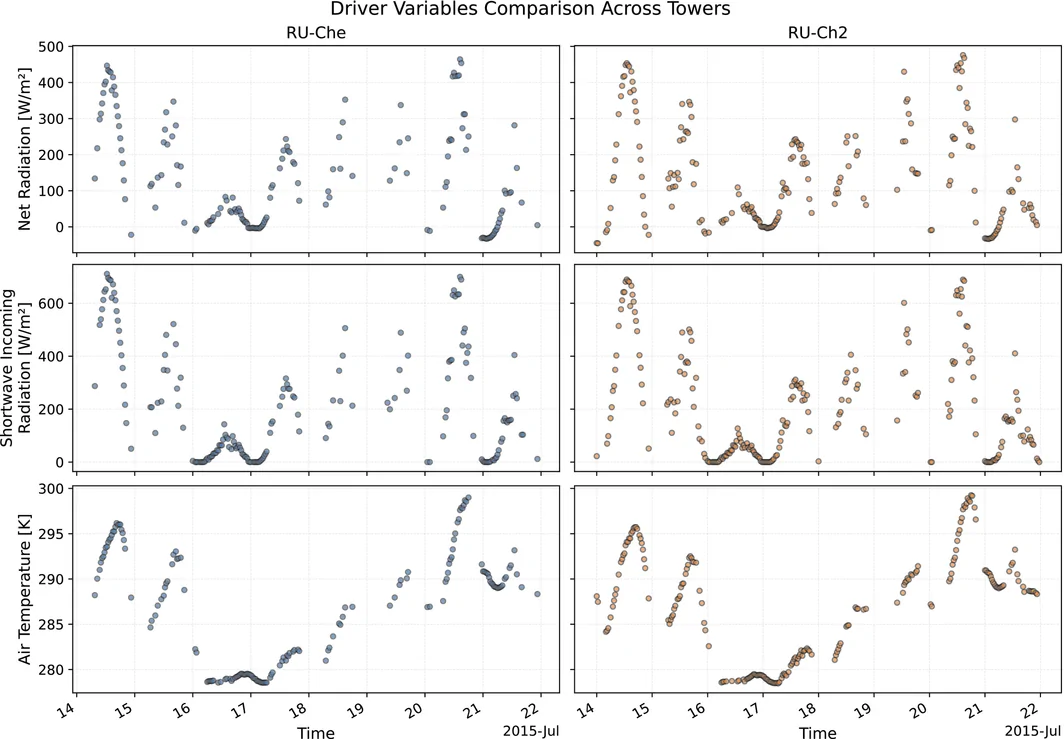
Measured time series of environmental driver variables at RU‐Che and RU‐Ch2 (columns): Net radiation (top row), shortwave incoming radiation (middle row) and air temperature (bottom row).

The sensible heat flux is modeled using net radiation and air temperature as driver variables, whereas the ${\mathrm{CO}}_{2}$ flux (NEE) is modeled using shortwave incoming radiation and air temperature. The corresponding time series for these driver variables are shown in Figure 8. The close agreement between measurements from the two towers corroborates the assumption (a) of spatial homogeneity of the environmental drivers required in Section 2.2.

#### 3.2.1 Cherskii Site: Sensible Heat Flux

**FIGURE 9:**
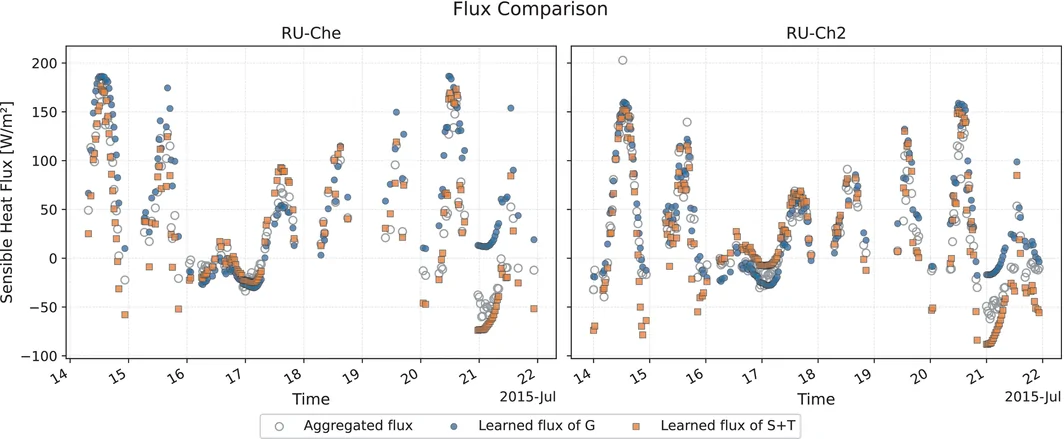
Learned sensible heat flux for grass (blue circles) and shrubs and trees (orange squares) against the measured aggregated flux (hollow gray circles) for towers RU‐Che (left) and RU‐Ch2 (right). The decomposition reveals distinct diurnal amplitudes for each land cover class at both towers, and the FLUGS‐decomposed sensible heat fluxes are consistent between the two independent towers observing the same landscape.

The learned sensible heat fluxes show good overall agreement between the two Cherskii towers, as illustrated in Figure 9. Quantitative diagnostics confirm a high‐quality decomposition at both sites. For RU‐Che, the regression performs particularly well, with $R^{2}=0.975$ and only mild autocorrelation in the residuals (DuWa = 1.240). RU‐Ch2 performs comparably, with $R^{2}=0.964$ and DuWa = 1.353. The residuals depart from normality at both towers (ShWi‐p = $7.7\times 10^{-3}$ at RU‐Che and $1.8\times 10^{-6}$ at RU‐Ch2). As explained below, the remaining differences between the towers may point to distinct sampling characteristics rather than limitations of FLUGS.

**FIGURE 10:**
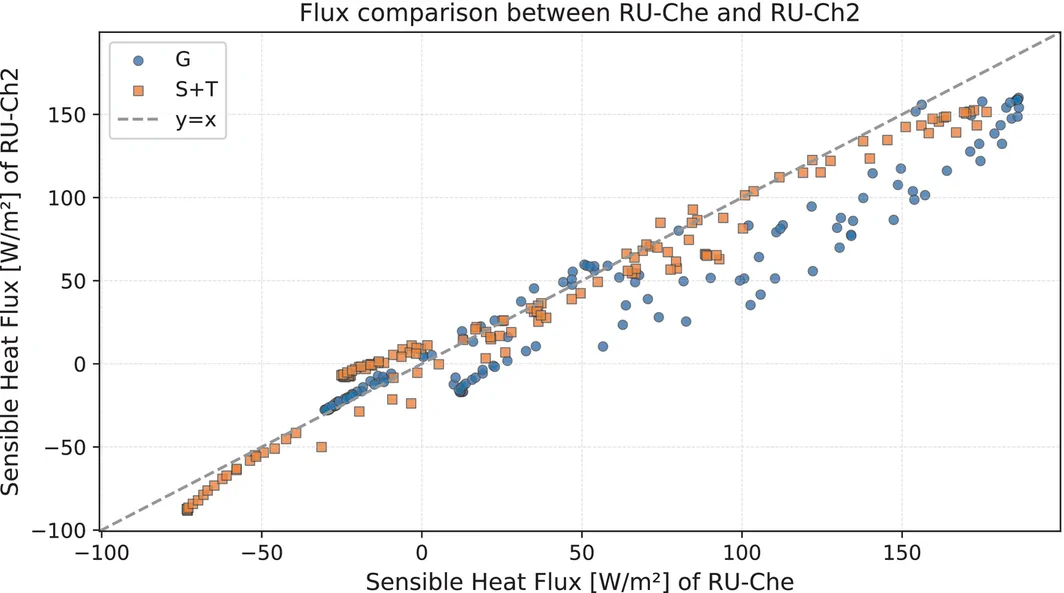
Cross‐tower scatter plot of FLUGS‐learned sensible heat fluxes for grass (blue circles) and shrubs‐and‐trees (orange squares). The dashed line indicates perfect agreement (y=x). The shrubs‐and‐trees class shows near‐perfect cross‐tower consistency, while the grass class exhibits a systematic offset. The two towers yield the similar land‐cover‐specific sensible heat fluxes.

To test whether towers observing different fractional compositions of the same landscape yield consistent LCC‐specific fluxes, we compare the FLUGS outputs from each tower in a cross‐tower scatter plot. The LCC‐specific comparison, shown in Figure 10, reveals a generally strong correlation between the two towers. An especially high correspondence for the shrubs‐and‐trees LCC was found. The flux decompositions for this class align almost perfectly between RU‐Che and RU‐Ch2. The grass LCC exhibits a slight but systematic offset, with consistently smaller sensible heat fluxes at RU‐Ch2.

The grass LCC bias is consistent with the known microtopographic and hydrological differences at the site. As an active floodplain, the terrain surrounding the towers is very flat, but even minor elevation gradients of just 1–2 m lead to a lateral redistribution of water, and the formation of dry ridges as well as inundated depressions. Tall vegetation tends to occur in naturally dry locations with lower water tables which were not strongly affected by the drainage; therefore conditions in these areas are similar across the two tower footprints. The lower lying areas, on the other hand, saw a severe change in the water table within the drainage area surrounding tower RU‐Che, followed by shifts in vegetation status, energy budgets and carbon cycling. Accordingly, the simplified land cover classification applied here aggregates different types of grassland and tussock tundra into a single class “grass,” while in reality conditions differ in the footprints of RU‐Che and RU‐Ch2. The RU‐Ch2 footprint contains extensive wet cotton grass meadows as well as a generally higher Bowen‐Ratio compared to the drier grassy and tussock areas surrounding RU‐Che, as also reported by Göckede et al. (2017). Consequently, the sensible heat flux at RU‐Ch2 is comparatively smaller than that of RU‐Che at a given net radiation as more available energy is converted into latent rather than sensible heat flux at the wetter site (not shown here). The observed differences therefore might partially violate the assumption (c) of ecosystem similarity but also reflect real ecological and hydrological contrasts rather than model bias.

**FIGURE 11:**
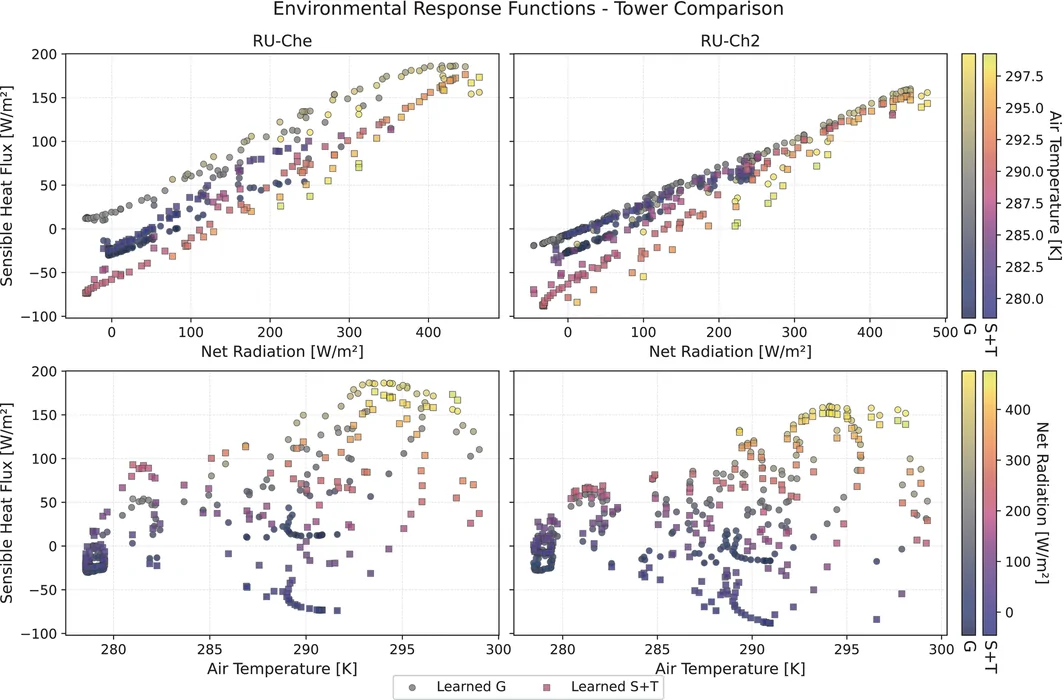
Learned environmental response functions for sensible heat flux: Grass (circles) and shrubs and trees (squares) for RU‐Che (left column) and RU‐Ch2 (right column). Upper panels show the flux–net‐radiation relationship; lower panels show the flux–temperature relationship. Marker colors indicate dependence on the secondary driver variable. The consistency of ERF shapes across towers confirms that FLUGS recovers physically meaningful driver–flux relationships from independent observations.

Figure 11 demonstrates that FLUGS recovers the ERFs for each LCC. For both towers, the upper panels show a coherent increase in sensible heat flux with net radiation, with distinct slopes for grass and shrubs‐and‐trees classes that reflect differences in vegetation structure. The color gradients indicate that these relationships are modulated by air temperature, particularly during periods of low radiative forcing in the grass LCC of tower RU‐Ch2. The lower panels reveal more complex patterns, where sensible heat flux varies with air temperature in loop‐like structures that reflect the diurnal forcing cycle. These cycles arise naturally from asymmetries in radiative input and differences in surface heating. The consistency of these response functions across towers, together with their systematic differences between land cover types, indicates that FLUGS recovers physically interpretable and ecologically meaningful driver‐flux relationships under real field conditions.

#### 3.2.2 Cherskii Site: CO_2_ Flux

**FIGURE 12:**
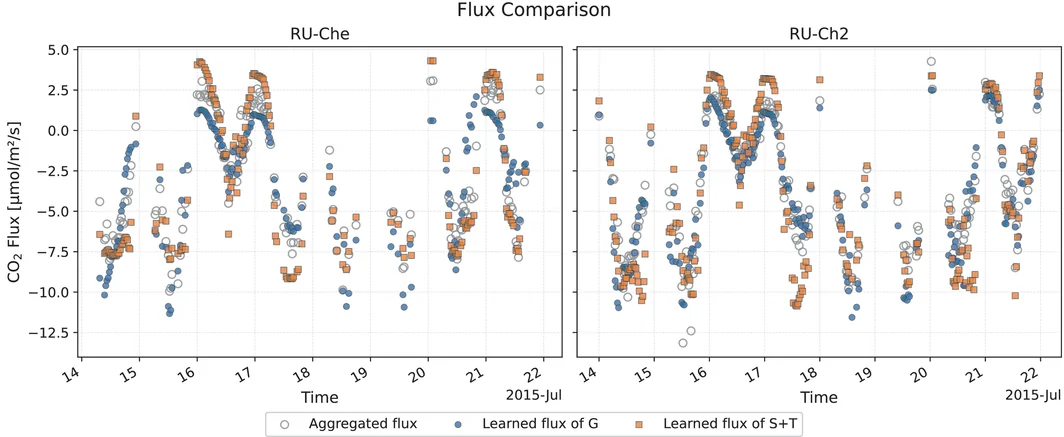
Cross‐tower consistency of FLUGS‐decomposed ${\mathrm{CO}}_{2}$ fluxes. Learned ${\mathrm{CO}}_{2}$ flux for grass (blue circles) and shrubs‐and‐trees (orange squares) against the measured aggregated flux (hollow gray circles) for RU‐Che (left) and RU‐Ch2 (right). The decomposition captures the expected diurnal pattern of daytime uptake and nighttime respiration for both land cover classes.

Figure 12 shows the learned ${\mathrm{CO}}_{2}$ fluxes for the two Cherskii towers. As in Section 3.2.1, fluxes are consistent across the towers with only a slight systematic offset due to local differences in hydrological conditions.

**FIGURE 13:**
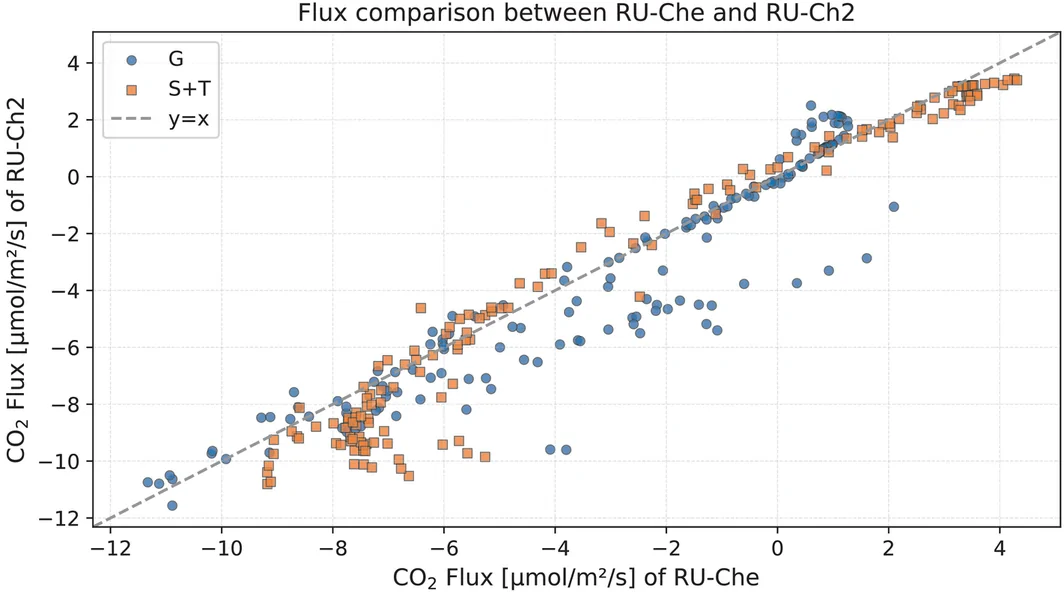
Cross‐tower scatter plot of FLUGS‐learned ${\mathrm{CO}}_{2}$ fluxes for grass (blue circles) and shrubs‐and‐trees (orange squares). The dashed line indicates perfect agreement. Shrubs‐and‐trees fluxes are highly consistent across towers. The grass class shows a systematic offset toward more positive (less uptake) values at RU‐Che. Both towers yield largely consistent land‐cover‐specific ${\mathrm{CO}}_{2}$ fluxes.

**FIGURE 14:**
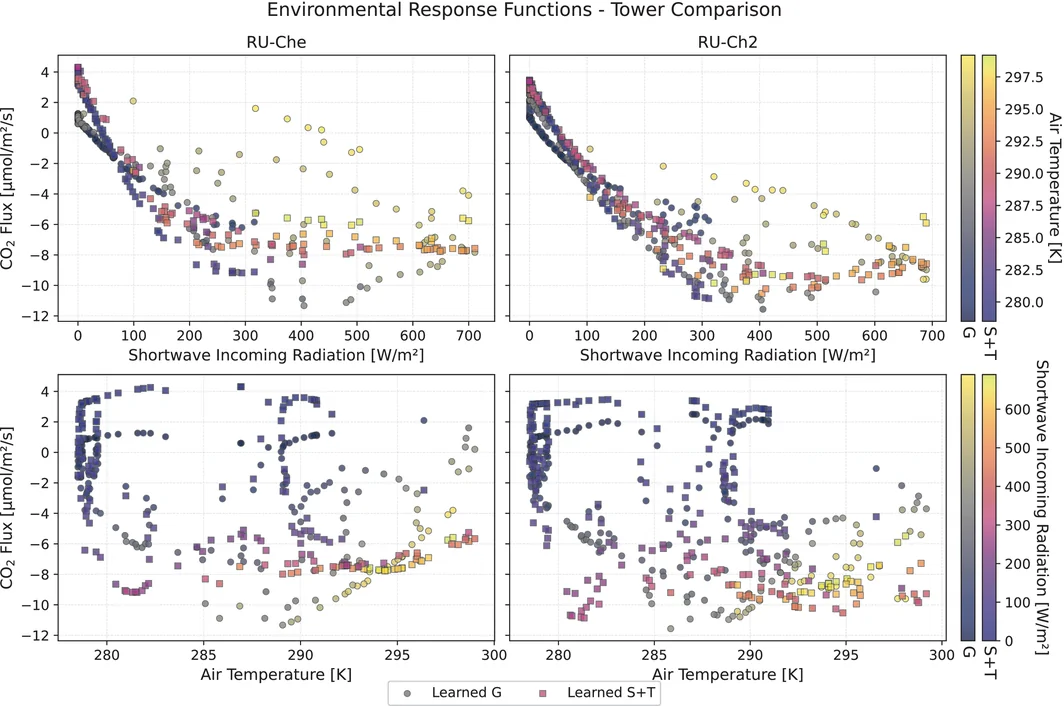
Learned environmental response functions for ${\mathrm{CO}}_{2}$ flux: Grass (circles) and shrubs‐and‐trees (squares) for RU‐Che (left column) and RU‐Ch2 (right column). Upper panels show the flux–shortwave‐radiation relationship; lower panels show the flux–temperature relationship. Marker colors indicate the secondary driver value. ERF shapes are consistent between towers at low driver values, with divergence at higher values.

As for sensible heat, we assess cross‐tower consistency of the ${\mathrm{CO}}_{2}$ decomposition by plotting the learned fluxes from RU‐Che against those from RU‐Ch2. The small systematic bias is corroborated by Figure 13. Specifically, the reduced ${\mathrm{CO}}_{2}$ uptake at RU‐Che likely reflects higher soil respiration and moisture limitation, as also reported in Bolek et al. (2025). The learned environmental response functions in Figure 14 adequately demonstrate the ecosystem similarity at the two sites for low temperature and low radiation but show the aforementioned offset for higher values, which supports the interpretation that RU‐Che exhibits more positive fluxes consistent with drier soils and higher daytime temperatures resulting in increased soil respiration which agrees with the findings of Göckede et al. (2019).

The responses to air temperature show clear diurnal cycles, with loop‐like structures that reflect morning–afternoon asymmetries in radiative forcing and surface heating. These patterns are recovered consistently for both towers and both vegetation classes, indicating that FLUGS captures realistic submeso‐scale drivers of ${\mathrm{CO}}_{2}$ exchange.

Including more explanatory driver variables can, in principle, strengthen these response relationships, although doing so increases the risk of overfitting when data are limited. The drivers chosen here are spatially homogeneous for the majority of EC sites. However, special attention needs to be given to drivers such as soil temperature or soil water content. In certain ecosystems, those are often spatially heterogeneous and provide limited gain for the training of FLUGS. The present results suggest that even with a modest driver set, FLUGS extracts coherent and ecologically interpretable ${\mathrm{CO}}_{2}$ flux patterns across the heterogeneous Cherskii landscape. This conclusion is backed with additional sensitivity studies to be found in Appendix D.

## 4 Conclusion

Spatial flux decomposition offers a promising pathway to increase the information content that can be extracted from existing EC measurements. By separating aggregated fluxes into land‐cover‐specific components, FLUGS dissects a single EC measurement into multiple land‐cover contributions, effectively increasing the dimensionality of the observational record and broadening the information available for evaluating, constraining and benchmarking land‐surface models. Especially in regions of low data coverage, FLUGS has the potential to improve data‐driven upscaling models by adding virtual EC towers to the network.

In the process, FLUGS learns the ERFs using KRR without any process knowledge and pre‐assumptions on the ecosystem or functional relationship to the environmental controls. As a convex optimization method, KRR and by extension FLUGS, guarantees a unique global optimum. Unlike Neural Networks or Boosted Regression Trees, it is free from convergence issues and the resulting ERFs are continuous by construction. The inherent regularization in KRR also allows it to handle highly correlated driver variables without overfitting. Furthermore, KRR has well‐established mathematical properties that facilitate transparent interpretation of model behavior, in contrast to the largely opaque nature of Neural Networks.

FLUGS' capability shifts the design considerations for future flux towers. Instead of prioritizing spatial homogeneity by minimizing footprint size, it becomes advantageous to instrument heterogeneous landscapes with taller towers that sample a broader area. Such tower deployments combined with FLUGS would provide richer ecological gradients and make more efficient use of field resources, ultimately strengthening the observational basis for understanding land‐atmosphere exchanges.

From the perspective of a site's manager, integrating FLUGS into their data processing pipeline is straightforward: the requirements are a land cover map roughly the size of the expected mean footprint extent, and processed flux data with quality flags but no gap‐filling. Quality flag filtering is performed internally by FLUGS. Since FLUGS is an open‐source software package, it can be installed and executed on a local computer. Furthermore, due to FLUGS' inherent modularity, the framework is easily automated and integrated into existing data streams. Nevertheless, expert knowledge is required for setting the analysis time window to comply with the ecosystem stationarity assumption. Ideally, the training period is as long as possible but without significant changes in the ecosystem due to, e.g., management, disturbances or succession. Also, the user chooses the environmental drivers. In general, the more drivers provided to the FLUGS framework, the better; however, special care should be taken to ensure compliance with the assumed spatial homogeneity of the drivers.

The present study validates FLUGS at a single site. The Cherskii site is, however, an exceptional case: two independently operated EC towers in the same heterogeneous landscape provide a rare cross‐tower consistency check without requiring external ground truth. Extending the evaluation to additional sites, plant functional types, and climate classes is the natural next step and is the subject of ongoing work.

## Author Contributions

**Mark Schlutow:** conceptualization, methodology, software, data curation, formal analysis, validation, visualization, writing – original draft, writing – review and editing. **Ray Chew:** conceptualization, writing – original draft, writing – review and editing, methodology, validation, visualization, software, formal analysis, data curation. **Mathias Göckede:** writing – review and editing, project administration, resources, supervision, funding acquisition.

## Funding

This research has been supported by the European Research Council, H2020 European Research Council (grant no. 951288) and the Deutsche Forschungsgemeinschaft (grant no. 235221301).

## Conflicts of Interest

The authors declare no conflicts of interest.

## Figures and Tables

**FIGURE A1:**
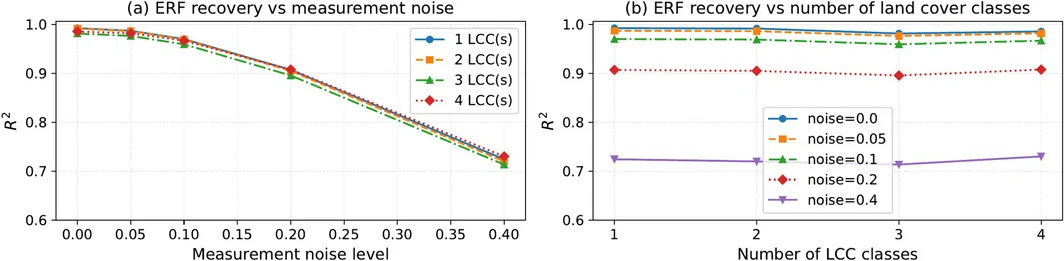
Sensitivity of FLUGS spatial decomposition to increasing measurement noise across different LCC configurations in the synthetic data experiment.

**FIGURE A2:**
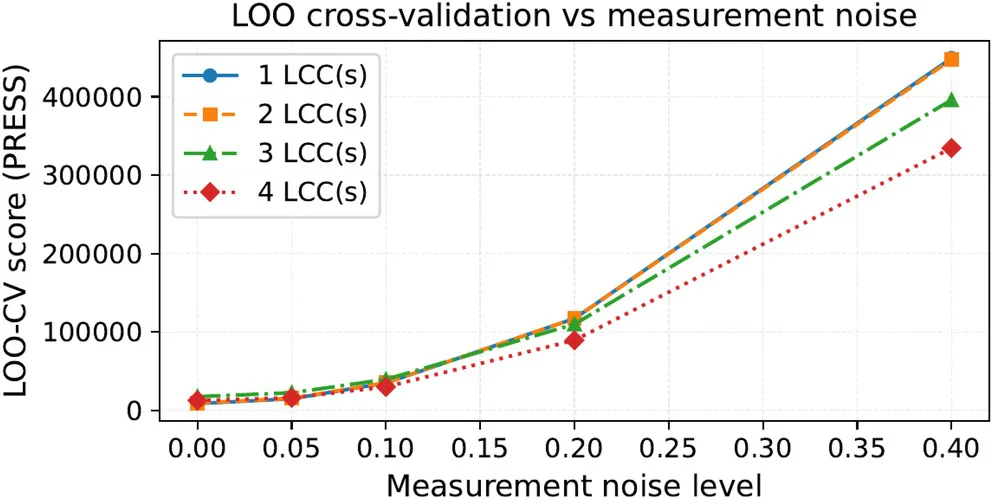
PRESS scores for the synthetic data experiment for different noise and LCC configurations; see accompanying text for more details.

## Data Availability

The data that support the findings of this study are openly available in Zenodo at https://zenodo.org/records/17733779 (Schlutow and Chew 2025b). The exact version of FLUGS used to produce the results used in this paper is archived on https://zenodo.org/records/17733284 (Schlutow and Chew 2025c). The source code of FLUGS is openly available at https://github.com/SchlutowSM2Group/FLUGS.

## Endnotes

^1^

https://github.com/SchlutowSM2Group/BLDFM.

^2^

https://www.licor.com/products/eddy‐covariance/eddypro.

## Appendix A

### Useful Formulas and Identities

This appendix contains a collection of formulas and identities that are needed for the derivation of the FLUGS method.

1. Visick (2000) proved that the Hadamard product (∘) between two N×N square matrices of equal size X and Y may be expressed as a principal submatrix of the Kronecker product (⊗),

(A1)
$$X\circ Y=P^{T}\left(X⊗Y\right)P,$$

where P is the selection matrix. The Kronecker product results in a block matrix where every element of the first matrix is multiplied with the entire second matrix. P is a $N^{2}\times N$ partial permutation matrix of zeros and ones with the following useful properties: The Diag operator, which returns the diagonal elements of a square matrix in a vector, can be rewritten in terms of the selection matrix as a linear operation,

(A2)
$$\begin{array}{r} \text{Diag}\left(X\right)=P^{T}\;\mathrm{vec}\left(X\right). \end{array}$$

Here, the $\mathrm{vec}\left(\cdot \right)$ operator rearranges an I×N matrix into an IN×1 column vector. Furthermore, the selection matrix acts on an N vector x as

(A3)
$$\begin{array}{r} P\;x=\mathrm{vec}\left(\mathrm{diag}\left(x\right)\right) \end{array}$$

with diag the operator entering a vector and returning the matrix of almost all zeros but the vector's values on the diagonal. Eventually, $P^{T}P=I$.

2 Vectorization and Kronecker product identities

(A4)
$$\begin{array}{ccc} \mathrm{vec}\left(\mathrm{XYZ}\right) & = & \left(Z^{T}⊗X\right)\;\mathrm{vec}\left(Y\right), \end{array}$$

(A5)
$$\begin{array}{ccc} {\left(X⊗Y\right)}^{T} & = & X^{T}⊗Y^{T} \end{array},$$

(A6)
$$\begin{array}{ccc} \left(X⊗Y\right)\left(U⊗V\right) & = & \left(\mathrm{XU}\right)⊗\left(\mathrm{YV}\right) \end{array}.$$

3 The Sherman‐Morrison‐Woodbury formula

(A7)
$$\begin{array}{c} \begin{array}{r} {\left(X^{-1}+U^{T}Y^{-1}U\right)}^{-1}U^{T}Y^{-1}=XU^{T}{\left(\mathrm{UX}U^{T}+Y\right)}^{-1}. \end{array} \end{array}$$

## Appendix B

### Derivation of the FLUGS Method

This appendix provides a detailed derivation of the FLUGS inversion method. Starting from the predictive model Equation (4) of the main text presented in Section 2.2 (Flux inversion with multitask multivariate kernel ridge regression), we derive a cost function and a linear model using ridge regression. This model is then generalized to operate in an infinite‐dimensional feature space by the “Kernel Trick.”

### Linear Model and Ridge Regression

This subsection formulates the flux inversion as a standard linear regression problem with regularization, expressing the unknown per‐class fluxes in terms of environmental drivers and footprint weights.

The prime objective in deriving the optimization task for the flux inversion is to capture the temporal dynamics of the scaling factors through environmental response functions (ERFs). ERFs link environmental driver variables to surface fluxes, applying the assumption of ecosystem similarity (Assumption c); see Section 2.2 (Flux inversion with multitask multivariate kernel ridge regression) for further elaboration. A simple but universal ERF may be provided by a linear model, for example,

(A8)
$$\begin{array}{r} a_{\text{in}}=\sum_{p=1}^{P}b_{\mathrm{ip}}x_{\mathrm{pn}} \end{array},$$

where p indexes the basis functions, for example, polynomials, that span the feature space and x represents functions of environmental driver variables, such as air temperature T, air humidity, shortwave incoming radiation, and PAR, that are usually measured by the EC tower and are assumed to be the same for each LCC at a given timestamp. The coefficients $b_{\mathrm{ip}}$ are the unknowns that are estimated by solving the optimization problem.

P is related to the degree of the polynomials and the number of environmental driver variables chosen by the user. Illustrating the concept, if we were to invert, for example, CO_2_ fluxes, a design matrix $\left(x_{\mathrm{pn}}\right)$ of second‐degree polynomials might look as follows:

$$\begin{array}{c} \begin{array}{r} X^{T}=\left(\begin{array}{cccccc} 1 & T_{1} & {\mathrm{PAR}}_{1} & T_{1}^{2} & T_{1}\;{\mathrm{PAR}}_{1} & {\mathrm{PAR}}_{1}^{2}\\ 1 & T_{2} & {\mathrm{PAR}}_{2} & T_{2}^{2} & T_{2}\;{\mathrm{PAR}}_{2} & {\mathrm{PAR}}_{2}^{2}\\ \vdots & \vdots & \vdots & \vdots & \vdots & \vdots \\ 1 & T_{N} & {\mathrm{PAR}}_{N} & T_{N}^{2} & T_{N}\;{\mathrm{PAR}}_{N} & {\mathrm{PAR}}_{N}^{2} \end{array}\right) \end{array}. \end{array}$$

Here, P=6. Note that the linear method is indeed capable of modeling nonlinear response functions. For later reference, (A8) may be written in matrix notation as

(A9)
$$\begin{array}{r} A=\mathrm{BX}, \end{array}$$

where $A=\left(a_{\text{in}}\right)$ and $B=\left(b_{\mathrm{ip}}\right)$. Inserting the linear ansatz above into the predictive model Equation (4) of the main text yields,

$$\begin{array}{r} q_{n}=\sum_{i=1}^{I}\sum_{p=1}^{P}w_{\mathrm{ni}}b_{\mathrm{ip}}x_{\mathrm{pn}} \end{array},$$

which we write in matrix notation as

$$\begin{array}{r} q=\text{Diag}\left(\mathrm{WBX}\right). \end{array}$$

With (A2) and (A4), this expression can be rearranged in more tractable form such that

(A10)
$$\begin{array}{r} q=P^{T}\left(X^{T}⊗W\right)\;\mathrm{vec}\left(B\right)=\tilde{X}\;b \end{array}.$$

where $\tilde{X}=P^{T}\left(X^{T}⊗W\right)$ the weighted and modified design matrix and $b=\mathrm{vec}\left(B\right)$ the parameter vector. The right‐hand side of (A8) represents the standard form of a linear model.

As the environmental driver variables are potentially correlated, we employ ridge regression, the least‐squares method with regularization, to mitigate multicollinearity. We write the cost function as follows:

$$\begin{array}{r} L\left(b\right)=∥\hat{q}-\tilde{X}\;b{∥}^{2}+\lambda ∥b{∥}^{2} \end{array}.$$

Here, λ is the regularization parameter that is considered a hyperparameter. To establish a distinction with the modeled fluxes q, the hatted $\hat{q}$ denotes the vector of measured EC fluxes.

To minimize the cost function, we take the gradient with respect to the parameter vector b and set it to zero, which may be written as:

$$\begin{array}{r} \frac{\partial L}{\partial \;b}=-2{\tilde{X}}^{T}\left(\hat{q}-\tilde{X}\;b\right)+2\lambda \;b\overset{!}{=}0 \end{array}.$$

Solving for the parameter vector yields

(A11)
$$\begin{array}{r} b={\left({\tilde{X}}^{T}\tilde{X}+\lambda I_{N}\right)}^{-1}{\tilde{X}}^{T}\;\hat{q} \end{array}$$

with $I_{N}$ the N×N identity matrix.

Vectorizing (A9) with identity (A4) results in

(A12)
$$\begin{array}{rr} \mathrm{vec}\left(A\right) & =\left(X^{T}⊗I_{I}\right)\;b \end{array},$$

where $I_{I}$ is the I×I identity matrix. Substituting with (A11) provides an expression for the matrix of scaling factors and completes the derivation of the linear model.

### Multitask Kernel Ridge Regression

This subsection generalizes the linear model to a nonparametric setting using the kernel trick, which allows FLUGS to learn arbitrary nonlinear ERFs while retaining a closed‐form, globally optimal solution.

The linear method derived above is the starting point for the introduction of the Multitask Kernel Ridge Regression, which is at the core of FLUGS. The method introduced in Section 2.2 (Flux inversion with multitask multivariate kernel ridge regression) fits naturally within multitask machine learning, as the ERFs of all LCCs are estimated in a single, coupled optimization. Here, we manipulate the findings of the previous section to obtain the inner product of the feature space, which is subsequently generalized by applying the “Kernel Trick.” For an in‐depth introduction to kernel methods, we refer the interested reader to Bishop (2006) and Álvarez et al. (2012).

Using a version of the Sherman‐Morrison‐Woodbury formula (A7), we can rearrange (A11), resulting in the following dual representation:

(A13)
$$\begin{array}{r} b={\tilde{X}}^{T}{\left(\tilde{X}{\tilde{X}}^{T}+\lambda I_{N}\right)}^{-1}\;\hat{q} \end{array}$$

Exploiting identities (A5) and (A6), plugging in (A13), we may rewrite (A12) as

(A14)
$$\begin{array}{c} \begin{array}{r} \mathrm{vec}\left(A\right)=\left(X^{T}X⊗W^{T}\right)P{\left[P^{T}\left(X^{T}X⊗WW^{T}\right)P+\lambda I_{N}\right]}^{-1}\;\hat{q} \end{array}. \end{array}$$

Recognizing that only inner products of the feature matrix occur in (A14), we can now apply the “Kernel Trick” by replacing any instance of $X^{T}X$ by its feature space generalization, the kernel matrix K, which gives us

(A15)
$$\begin{array}{c} \begin{array}{r} \mathrm{vec}\left(A\right)=\left(K⊗W^{T}\right)P{\left[P^{T}\left(K⊗WW^{T}\right)P+\lambda I_{N}\right]}^{-1}\;\hat{q} \end{array}. \end{array}$$

When we apply (A1), (A3), and (A4), we can eventually simplify the expression for the scaling factors to

$$\begin{array}{r} A=W^{T}\mathrm{diag}\left({\left(K\circ WW^{T}+\lambda I_{N}\right)}^{-1}\;\hat{q}\right)K \end{array}.$$

The multitask Kernel Ridge Regression formulation completes the full derivation of the FLUGS method.

## Appendix C

### Synthetic Environmental Response Function

The synthetic net ecosystem exchange (NEE) environment response function for each land cover class (LCC) is based on Runkle et al. (2013) and computed as follows:

$$\begin{array}{c} \begin{array}{r} \mathrm{NEE}\left(\mathrm{PAR},T\right)=R_{10}Q_{10}^{\left(T-T_{\mathrm{ref}}\right)/\gamma }-\frac{P_{\max}\alpha \mathrm{PAR}}{P_{\max}+\alpha \mathrm{PAR}} \end{array}, \end{array}$$

with the parameters given in Table A1.

**TABLE A1:** Parameters used to generate the environment response function and driver variables for the synthetic data experiment in Section 2.3.1 (Corroboration by synthetic data in a virtual experiment).

LCC	$P_{\max}\left[\mathrm{\mu g}.m^{-2}.s^{-1}\right]$	$\alpha \left[\mathrm{\mu g}.\mathrm{\mu mo}l^{-1}\right]$	$R_{10}\left[\mathrm{\mu g}.m^{-2}.s^{-1}\right]$	$Q_{10}$		
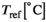
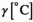
0	400	1.1	30	2.0	15	5
1	200	0.4	50	1.5	15	10

The diurnally varying driver variable PAR is generated as follows:

$$\mathrm{PAR}\left(h\right)=\max\left(0,-{\mathrm{PAR}}_{\max}\cos\;\left(\frac{\pi }{12}h\right)\right)+\varepsilon_{\mathrm{PAR}},$$

where $\varepsilon_{\mathrm{PAR}}∼N\left(0,\sigma_{\mathrm{PAR}}^{2}\right)$ and $\sigma_{\mathrm{PAR}}=0.1\;{\mathrm{PAR}}_{\max}$. PAR is rectified to zero to avoid negative irradiance. The temperature T is given by

$$T\left(h\right)=\left[T_{\mathrm{amp}}\;\sin^{4}\;\left(\frac{\pi }{24}\left(h-2\right)\right)+5.0\right]+\varepsilon_{T},$$

with $\varepsilon_{T}∼N\left(0,\sigma_{T}^{2}\right)$ and $\sigma_{T}=0.1\;T_{\mathrm{amp}}$. The fractional hours from a given start time, h, is calculated as

$$h=\left(\mathrm{day}-1\right)\cdot 24+\text{hour}+\frac{\text{minute}}{60},$$

where day, hour and minute are durations since a specified start time.

## Appendix D

### FLUGS Sensitivity Studies

This appendix provides sensitivity studies and validations for the experiments found in Section 3 (Results and Discussion).

### A Synthetic Sensitivity Sweep

In the first study, we extend the synthetic experiment of Section 3.1 (Virtual experiment with synthetic data) for sweeps over:

- Measurement noise: 0%, 5%, 10%, 20%, 40% of the flux magnitude
- Number of LCCs: 1, 2, 3, 4

Note that flux noise is added as white noise directly to the aggregated tower flux. We intentionally avoid adding noise to the environmental drivers (PAR, Ts). Adding noise to the drivers would artificially increase the spread of observations in driver space, providing the kernel regression with more coverage of the surface responses. This would paradoxically improve the $R^{2}$ scores for the wrong reasons. Therefore, flux noise is the appropriate challenge, as it degrades the signal quality without artificially inflating the effective dimensionality of the driver space.

The results in Figure A1 show that the $R^{2}$ across all LCC configurations remains above 0.89 up to 20% noise, and above 0.71 even at 40% noise. Performance is stable across one to four LCCs, confirming generality beyond the two‐class scenario.

### Cross‐Validation and Potential Overfitting

In this study, we compute the Leave‐One‐Out Cross‐Validation (LOO‐CV) score analytically from the eigendecomposition of the weighted kernel matrix in Equation (6) of the main text. The LOO‐CV score is the predicted residual error sum of squares (PRESS statistic),

(A16)
$$\mathrm{LOO}=\sum_{n=1}^{N}{\left(\frac{r_{n}}{1-h_{\mathrm{nn}}}\right)}^{2},$$

where $r_{n}$ is the residual of the ith data point and $h_{\mathrm{nn}}$ are the diagonal elements computed from an eigenvalue decomposition. This is exact for the ridge regression problem in FLUGS and requires no refitting. Figure A2 shows that the LOO‐CV scores for the synthetic data experiment increase monotonically with noise, confirming that the score tracks true prediction error rather than overfitting the data.

To assess potential overfitting in the Cherskii case study under realistic data constraints, we employ two complementary diagnostics. First, we compare the LOO‐CV score against the training residual sum of squares ${\mathrm{SS}}_{\mathrm{res}}=\sum_{n}r_{n}^{2}$ for both learned sensible heat flux and CO_2_ at each tower. The ratio $\mathrm{LOO}/{\mathrm{SS}}_{\mathrm{res}}$ quantifies the inflation of cross‐validated prediction error relative to training error: A ratio near unity indicates that the regularization effectively prevents high‐leverage points from dominating the fit.

Second, we translate the PRESS statistic into a cross‐validated coefficient of determination, $R_{\mathrm{CV}}^{2}=1-\text{PRESS}/{\mathrm{SS}}_{\mathrm{tot}}$, where ${\mathrm{SS}}_{\mathrm{tot}}=\sum_{n}{\left(y_{n}-\bar{y}\right)}^{2}$ is the total sum of squares, to allow direct comparison with the scores from the experiments in the main text, $R_{\text{train}}^{2}=1-{\mathrm{SS}}_{\mathrm{res}}/{\mathrm{SS}}_{\mathrm{tot}}$.

Results from Table A2 show that the cross‐validated $R_{\mathrm{CV}}^{2}$ tracks the training $R_{\text{train}}^{2}$ closely, with relative drops below 2% across all four subsets. This close agreement is the primary evidence that the learned ERFs generalize rather than overfit the observational data. The $\mathrm{LOO}/{\mathrm{SS}}_{\mathrm{res}}$ ratios (between 1.20 and 1.45) are consistent with the regularized fit's modest effective dimensionality of ∼8%–15% of the N observations. These ratios therefore reflect the expected leave‐one‐out penalty rather than a symptom of overfitting. Finally, we compute the Durbin–Watson and Shapiro–Wilk statistics on the PRESS residuals $r_{n}/\left(1-h_{\mathrm{nn}}\right)$ to directly compare residual structure between training and cross‐validated predictions. As shown in Table A2, the diagnostics are virtually identical, confirming that the error structure remains stable when observations are held out.

**TABLE A2:** Comparison of training and cross‐validated diagnostics for the Cherskii towers across learned sensible heat flux (SHF) and CO_2_. The minimal drop between $R_{\text{train}}^{2}$ and $R_{\mathrm{CV}}^{2}$, modest error ratios, and near‐identical training and cross‐validated residual diagnostics confirm stable model generalization.

Metric	SHF Tower 1	SHF Tower 2	CO_2_ Tower 1	CO_2_ Tower 2
$R_{\text{train}}^{2}$	0.975	0.964	0.967	0.955
$R_{\mathrm{CV}}^{2}$	0.968	0.957	0.952	0.940
${\mathrm{SS}}_{\mathrm{res}}$	15751.1	20519.2	82.1	142.7
LOO‐CV (PRESS)	20375.7	24523.9	119.4	188.1
Ratio ($\mathrm{LOO}/{\mathrm{SS}}_{\mathrm{res}}$)	1.29	1.20	1.45	1.32
DuWa (train)	1.240	1.353	1.614	1.636
DuWa (CV)	1.277	1.373	1.604	1.690
ShWi‐p (train)	0.008	< 0.001	0.416	< 0.001
ShWi‐p (CV)	0.027	< 0.001	0.429	<0.001

## References

[gcb71031-bib-0001] Álvarez, M. A. , L. Rosasco , and N. D. Lawrence . 2012. “Kernels for Vector‐Valued Functions: A Review.” Foundations and Trends in Machine Learning 4, no. 3: 195–266. 10.1561/2200000036.

[gcb71031-bib-0002] Baldocchi, D. D. 2020. “How Eddy Covariance Flux Measurements Have Contributed to Our Understanding of Global Change Biology.” Global Change Biology 26, no. 1: 242–260. 10.1111/gcb.14807. 31461544

[gcb71031-bib-0003] Bishop, C. 2006. Pattern Recognition and Machine Learning. Springer.

[gcb71031-bib-0004] Bolek, A. , M. Schlutow , T. Yazbeck , N. Triches , M. Heimann , and M. Göckede . 2025. “Impact of Long‐Term Drainage on Carbon Fluxes in the High‐Latitude Permafrost Region.” Global Change Biology 31, no. 7: e70346. 10.1111/gcb.70346. 40662385 PMC12261280

[gcb71031-bib-0005] Chu, H. , X. Luo , Z. Ouyang , et al. 2021. “Representativeness of Eddy‐Covariance Flux Footprints for Areas Surrounding AmeriFlux Sites.” Agricultural and Forest Meteorology 301–302: 108350. 10.1016/j.agrformet.2021.108350.

[gcb71031-bib-0006] Crawford, B. , and A. Christen . 2015. “Spatial Source Attribution of Measured Urban Eddy Covariance CO2 Fluxes.” Theoretical and Applied Climatology 119, no. 3: 733–755. 10.1007/s00704-014-1124-0.

[gcb71031-bib-0007] Durbin, J. , and G. S. Watson . 1950. “Testing for Serial Correlation in Least Squares Regression: I.” Biometrika 37, no. 3/4: 409–428. 10.2307/2332391. 14801065

[gcb71031-bib-0008] Göckede, M. , T. Foken , M. Aubinet , et al. 2008. “Quality Control of CarboEurope Flux Data – Part 1: Coupling Footprint Analyses With Flux Data Quality Assessment to Evaluate Sites in Forest Ecosystems.” Biogeosciences 5, no. 2: 433–450. 10.5194/bg-5-433-2008.

[gcb71031-bib-0009] Göckede, M. , F. Kittler , M. J. Kwon , et al. 2017. “Shifted Energy Fluxes, Increased Bowen Ratios, and Reduced Thaw Depths Linked With Drainage‐Induced Changes in Permafrost Ecosystem Structure.” Cryosphere 11, no. 6: 2975–2996. 10.5194/tc-11-2975-2017.

[gcb71031-bib-0010] Göckede, M. , M. J. Kwon , F. Kittler , M. Heimann , N. Zimov , and S. Zimov . 2019. “Negative Feedback Processes Following Drainage Slow Down Permafrost Degradation.” Global Change Biology 25, no. 10: 3254–3266. 10.1111/gcb.14744. 31241797 PMC6851682

[gcb71031-bib-0011] Göckede, M. , C. Rebmann , and T. Foken . 2004. “A Combination of Quality Assessment Tools for Eddy Covariance Measurements With Footprint Modelling for the Characterisation of Complex Sites.” Agricultural and Forest Meteorology 127, no. 3: 175–188. 10.1016/j.agrformet.2004.07.012.

[gcb71031-bib-0012] Kittler, F. , I. Burjack , C. A. R. Corradi , et al. 2016. “Impacts of a Decadal Drainage Disturbance on Surface–Atmosphere Fluxes Of Carbon Dioxide in a Permafrost Ecosystem.” Biogeosciences 13, no. 18: 5315–5332. 10.5194/bg-13-5315-2016.

[gcb71031-bib-0013] Lawrence, D. M. , R. A. Fisher , C. D. Koven , et al. 2019. “The Community Land Model Version 5: Description of New Features, Benchmarking, and Impact of Forcing Uncertainty.” Journal of Advances in Modeling Earth Systems 11, no. 12: 4245–4287. 10.1029/2018MS001583.

[gcb71031-bib-0014] Levy, P. , J. Drewer , M. Jammet , et al. 2020. “Inference of Spatial Heterogeneity in Surface Fluxes From Eddy Covariance Data: A Case Study From a Subarctic Mire Ecosystem.” Agricultural and Forest Meteorology 280: 107783. 10.1016/j.agrformet.2019.107783.

[gcb71031-bib-0015] Metzger, S. , W. Junkermann , M. Mauder , et al. 2013. “Spatially Explicit Regionalization of Airborne Flux Measurements Using Environmental Response Functions.” Biogeosciences 10, no. 4: 2193–2217. 10.5194/bg-10-2193-2013.

[gcb71031-bib-0016] Moutahir, H. , M. Sulzer , R. Kiese , et al. 2026. “Matching Scales of Eddy Covariance Measurements and Process‐Based Modeling–Assessing Spatiotemporal Dynamics of Carbon and Water Fluxes in a Mixed Forest in Southern Germany.” Biogeosciences 23: 1719–1738. 10.5194/bg-23-1719-2026.

[gcb71031-bib-0017] Müller, S. , L. Schüler , A. Zech , and F. Heße . 2022. “GSTools v1.3: A Toolbox for Geostatistical Modelling in Python.” Geoscientific Model Development 15, no. 7: 3161–3182. 10.5194/gmd-15-3161-2022.

[gcb71031-bib-0018] Pirk, N. , K. Aalstad , E. S. Mannerfelt , et al. 2024. “Disaggregating the Carbon Exchange of Degrading Permafrost Peatlands Using Bayesian Deep Learning.” Geophysical Research Letters 51, no. 10: e2024GL109283. 10.1029/2024GL109283.

[gcb71031-bib-0019] Rey‐Sanchez, C. , A. Arias‐Ortiz , K. Kasak , et al. 2022. “Detecting Hot Spots of Methane Flux Using Footprint‐Weighted Flux Maps.” Journal of Geophysical Research: Biogeosciences 127, no. 8: e2022JG006977. 10.1029/2022JG006977. PMC9542288 36248720

[gcb71031-bib-0020] Rödenbeck, C. , S. Zaehle , R. Keeling , and M. Heimann . 2018. “How Does the Terrestrial Carbon Exchange Respond to Inter‐Annual Climatic Variations? A Quantification Based on Atmospheric CO_2_ Data.” Biogeosciences 15, no. 8: 2481–2498. 10.5194/bg-15-2481-2018.

[gcb71031-bib-0021] Runkle, B. R. K. , T. Sachs , C. Wille , E. M. Pfeiffer , and L. Kutzbach . 2013. “Bulk Partitioning the Growing Season Net Ecosystem Exchange of CO_2_ in Siberian Tundra Reveals the Seasonality of Its Carbon Sequestration Strength.” Biogeosciences 10, no. 3: 1337–1349. 10.5194/bg-10-1337-2013.

[gcb71031-bib-0023] Schlutow, M. , and R. Chew . 2025a. “BLDFM: The Boundary Layer Dispersion and Footprint Model.” *Zenodo*. 10.5281/zenodo.15487244.

[gcb71031-bib-0022] Schlutow, M. , and R. Chew . 2025b. Datasets for “FLUGS: Spatial Decomposition of Eddy Covariance Fluxes.” https://zenodo.org/records/17733779.10.1111/gcb.7103142657677

[gcb71031-bib-0024] Schlutow, M. , and R. Chew . 2025c. “FLUGS: Software Package for Spatial Decomposition of Eddy Covariance Fluxes.” 10.5281/zenodo.17733284.42657677

[gcb71031-bib-0025] Schlutow, M. , R. Chew , and M. Göckede . 2026. “The Boundary Layer Dispersion and Footprint Model: A Fast Numerical Solver of the Eulerian Steady‐State Advection‐Diffusion Equation.” Geoscientific Model Development 19: 6259–6272. 10.5194/gmd-19-6259-2026.

[gcb71031-bib-0026] Schlutow, M. , T. Stacke , T. Doerffel , P. K. Smolarkiewicz , and M. Göckede . 2024. “Large Eddy Simulations of the Interaction Between the Atmospheric Boundary Layer and Degrading Arctic Permafrost.” Journal of Geophysical Research: Atmospheres 129, no. 18: e2024JD040794. 10.1029/2024JD040794.

[gcb71031-bib-0027] Shapiro, S. S. , and M. B. Wilk . 1965. “An Analysis of Variance Test for Normality (Complete Samples).” Biometrika 52, no. 3–4: 591–611. 10.1093/biomet/52.3-4.591.

[gcb71031-bib-0028] Thum, T. , S. Caldararu , J. Engel , et al. 2019. “A New Model of the Coupled Carbon, Nitrogen, and Phosphorus Cycles in the Terrestrial Biosphere (QUINCY v1.0; Revision 1996).” Geoscientific Model Development 12, no. 11: 4781–4802. 10.5194/gmd-12-4781-2019.

[gcb71031-bib-0029] Tuovinen, J. P. , M. Aurela , J. Hatakka , et al. 2019. “Interpreting Eddy Covariance Data From Heterogeneous Siberian Tundra: Land‐Cover‐Specific Methane Fluxes and Spatial Representativeness.” Biogeosciences 16, no. 2: 255–274. 10.5194/bg-16-255-2019.

[gcb71031-bib-0030] Visick, G. 2000. “A Quantitative Version of the Observation That the Hadamard Product Is a Principal Submatrix of the Kronecker Product.” Linear Algebra and Its Applications 304, no. 1–3: 45–68. 10.1016/S0024-3795(99)00187-1.

[gcb71031-bib-0031] Wang, W. , K. J. Davis , B. D. Cook , M. P. Butler , and D. M. Ricciuto . 2006. “Decomposing CO_2_ Fluxes Measured Over a Mixed Ecosystem at a Tall Tower and Extending to a Region: A Case Study.” Journal of Geophysical Research: Biogeosciences 111, no. G2: G02005. 10.1029/2005JG000093.

[gcb71031-bib-0032] Xu, K. , S. Metzger , and A. R. Desai . 2017. “Upscaling Tower‐Observed Turbulent Exchange at Fine Spatio‐Temporal Resolution Using Environmental Response Functions.” Agricultural and Forest Meteorology 232: 10–22. 10.1016/j.agrformet.2016.07.019.

[gcb71031-bib-0033] Yazbeck, T. , M. Schlutow , A. Bolek , N.Y. Triches , E. Wahl , M. Heimann , and M. Göckede . 2025. “Quantifying Landcover‐Specific Fluxes Over a Heterogeneous Landscape Through Coupling UAV‐Measured Mixing ratios With a Large‐Eddy Simulation Model and Eddy‐Covariance Measurements.” Atmospheric Measurement Techniques 18: 6917–6932. 10.5194/amt-18-6917-2025.

